# Exploring the Involvement of Cytokines in Pediatric Patients Afflicted by Simple Febrile Seizures: A Case-Control Study

**DOI:** 10.7759/cureus.48083

**Published:** 2023-10-31

**Authors:** Vaibhav Vishal, Ashuma Sachdeva, Kapil Bhalla, Ankanksha Dureja, Sagar Tarte

**Affiliations:** 1 Biochemistry, Pandit Bhagwat Dayal Sharma Post Graduate Institute of Medical Sciences, Rohtak, IND; 2 Pediatrics, Pandit Bhagwat Dayal Sharma Post Graduate Institute of Medical Sciences, Rohtak, IND

**Keywords:** interleukin-6, hemogram parameters, interleukin-4, complex febrile seizures, anti-inflammatory cytokine

## Abstract

Aim: Most children under six with seizures experience febrile seizures (FS), which occur with a temperature of 38°C or higher and no apparent infectious or metabolic causes. FS has a multifaceted etiology, involving genetic and environmental factors. This study aimed to explore the connection between the cytokine system (part of the immune system related to inflammation) and FS to uncover potential relationships.

Method: This research was carried out on 50 patients experiencing FS and 25 patients experiencing only fever served as cases and controls, respectively. The patient’s blood was obtained under sterile circumstances from the antecubital/femoral vein as soon as feasible following the commencement of seizures. The complete hemogram analysis was done using the Mindray BC-5800 auto hematology analyzer (Mindray Medical International Limited, Shenzhen, China).

Result: The cases group had significantly higher interleukin-4 (IL-4) concentrations (292.85 pg/mL) than controls (81.04 pg/mL), indicating a statistically significant difference (p<0.05), respectively. In the current research, case participants had a significantly lower mean level of interleukin-6 (IL-6) than control participants. The average IL-6 concentration in cases was 28.00 pg/mL, whereas in controls was 93.09pg/mL. Patients with FS have an important function for the cytokine network to perform.

Conclusion: The findings showed a significant difference in cytokine concentrations between patients with FS and control subjects, highlighting a potential link between the cytokine system and FS. Additionally, lower levels of IL-6 in case participants suggest a complex role of cytokines in FS, emphasizing the importance of the cytokine network in this condition.

## Introduction

Seizures known as febrile seizure (FS) are the most prevalent kind of epilepsy in children and mainly strike children less than six years of age [1] who have a temperature of 38.0°C or greater with no other medical histories, such as CNS infection or metabolic abnormalities. The majority of FS are observed in children aged 18 to 24 months, with a significantly lower likelihood of occurrence in children under six months or over three years of age. Children in Japan and India are more likely to be affected by FS than those in the United States and Western Europe, where the prevalence is between 2% and 5% [2]. There is consistent evidence that males are more likely to have FS than females (the male-to-female ratio ranges from 1.1:1 to 2:1) [2]. Incidence rates of FS are highest in the winter months, when many children are sick with viral gastrointestinal diseases, and in the winter months, when more children are sick with viral upper respiratory infections. Treatment is usually not necessary for FS since most instances are mild and resolve on their own [2].

FS is often manifested on day one of a fever. Balosso et al. suggested that maximum temperature, rather than the rate of temperature increase, is a more important predictor of the onset of the first FS. The young brain's reaction to fever depends on its age. An increase in neuronal excitability throughout development puts a youngster at increased risk for FS. Seizures occur when the brain's temperature rises over its normal range, which causes changes in neuronal functioning and ion channels that affect neuronal firing and excitability. Seizures, as well as a number of neuropathological situations, such as ischemia and traumatic and excitotoxic brain traumas, cause a fast upregulation of the pleiotropic cytokine TNF-a in the CNS [3]. In Tutuncuoglu et al.'s research, individuals with FS during the acute phase had substantially higher levels of TNF-a in the cerebrospinal fluid than did patients in the control group [4]. Haspolat et al., on the other hand, found no variation in the quantities of tumor necrosis factor α in serum and cerebrospinal fluid in the control group and the children with FS [5]. In around 80% of instances of FS, fever is caused by a viral infection. However, both genetic and environmental factors have been implicated as contributors to the development of FS [6], which makes pinpointing a single cause difficult. Metabolic deregulatory pathways are thought to be activated by environmental factors. It has also been hypothesized that anemia caused by a lack of iron is a contributor to FS [7]. Cytokines are inflammatory mediators thought to have a role in FS [8]. Many different cells, both hematopoietic and non-hematopoietic, secrete soluble proteins called cytokines. They have a significant impact on both innate and adaptive immune responses and may experience changes in expression in several immunological, inflammatory, or infectious disease conditions [9]. In this situation, Kira et al. found that individuals with uncomplicated, sporadic FS had a considerably greater prevalence of the interleukin1B -31C/511T allele and homozygotes than control participants [10]. Similarly, while there was no discernible difference in the distribution of allele frequencies of the interleukin-1b promoter, interleukin-1b exon 5, interleukin-6 promoter, interleukin-8, interleukin-10, or the tumor necrosis factor α gene polymorphism (104 patients with FS and 143 healthy controls), Chou et al. reported that patients with FS were more likely to have the interleukin-1 receptor antagonist allele 1 homozygous [11,12].

While interleukin stimulates the production of CRP, serum amyloid A, fibrinogen, or hepcidin in hepatocytes, interleukin-6 (IL-6) also inhibits albumin formation, making it a pleiotropic cytokine. Rapid and temporary synthesis of IL-6 in response to infections and tissue injury supports host defense mechanisms such as the acute phase response, hematopoiesis, and immunological reactions [13].

The impact of cytokines, which can be both pro-inflammatory and anti-inflammatory, on immunological inflammatory diseases has been extensively researched. However, the significance of cytokines in the context of FS, which involves a neuroimmune response, remains inadequately explored. There is a notable gap in understanding how cytokines, particularly interleukin-4 (IL-4) and IL-6, may be involved in FS, especially in terms of recurrence. Thus, the primary objective of this study was to investigate the role of the cytokine system in patients with FS and explore the potential for a novel treatment approach targeting this system. The study aimed to ascertain whether the cytokine system plays a substantial role in the occurrence of FS and whether modulating this system could offer therapeutic benefits for children at risk of experiencing FS. By focusing on the cytokine system's involvement in FS, this research aimed to pave the way for innovative treatment strategies and enhance our understanding of the pathophysiology of FS.

## Materials and methods

This case-control study was carried out by the Department of Biochemistry and the Department of Pediatrics at Pandit Bhagwat Dayal Sharma Post Graduate Institute of Medical Sciences, Rohtak, India, from November 2022 to May 2023. The Institutional Ethics Committee of Pandit Bhagwat Dayal Sharma Post Graduate Institute of Medical Sciences/University of Health Sciences approved the study (approval number: IEC/TH/18/Biochem/05).

Study participants

According to the overall population of seizure and fever cases in the hospital, a total of 50 children who had FS were used as cases, while 25 children who only had a fever were used as controls.

Sample collection and procedure

In this case-control study, samples were obtained for both normal and special investigations, accompanied by a comprehensive medical history assessment. After obtaining informed consent from parents, blood samples of the children were collected, comprising 4 mL drawn from the antecubital/femoral vein using a red-capped blood collection tube. These samples were collected within a six-hour window from the onset of seizures or as early as possible, given feasibility constraints. Additionally, for ethical reasons, 2 mL of blood was drawn from the same vein using a purple-capped blood collection tube containing ethylenediaminetetraacetic acid (EDTA). The EDTA-tube blood samples were subjected to a complete hemogram analysis using a 5-cell counter, specifically, the Mindray BC-5800 auto hematology analyzer (Mindray Medical International Limited, Shenzhen, China). Furthermore, a peripheral blood film was prepared to examine the presence of malaria parasites.

Within an hour after being drawn, the blood in the red blood cell collecting tube was processed. After the blood had started to clot, it was spun at a speed of 2000 rpm for 10 minutes. The glucose levels in the separated serum were measured using a Randox auto analyzer (Randox Laboratories Ltd, Crumlin, UK) and a standard kit that same day, while the IL-6 and IL-4 levels were measured later, and the remainder was frozen at -70°C. Enzyme-linked immunosorbent assay (ELISA) was used to measure the IL-4 and IL-6 concentrations in the blood [14-19].

Statistical analysis

Statistical analysis was executed using SPSS Statistics version 25 (IBM Corp. Released 2017. IBM SPSS Statistics for Windows, Version 25.0. Armonk, NY: IBM Corp.), employing mean and standard deviation for continuous variables and numbers and percentages for categorical data. Descriptive statistics facilitated the assessment of frequencies and proportions. The difference in the variables between the two groups was calculated by applying Chi-square and Mann-Whitney U-tests.

## Results

Table 1 presents a comparison between the two groups, cases (n=50) and controls (n=25), across various characteristics. In terms of age, the cases have a lower mean age (2.73 years) compared to the controls (3.58 years). There was a higher proportion of males in the cases (27 out of 50) compared to the controls (14 out of 25). Interestingly, the cases have a slightly lower mean height (87.98 cm) compared to the controls (95.4 cm), but the difference in weight between the two groups was more pronounced, with cases having a lower mean weight (11.25 kg) compared to controls (13.28 kg). The weight-to-height ratio was quite similar in both groups.

**Table 1:** Demographic characteristics RBS: random blood sugar, Hb: hemoglobin, TLC: total leukocyte count, n: number

Characteristics		Case (n=50)	Control (n=25)
Age		2.734±1.40	3.58±1.21
Sex	Male	27	14
	Female	23	11
Height		87.98±12.02	95.4±9.78
Weight		11.24±2.60	13.28±2.31
Weight/height		14.55±2.11	14.53±1.10
Temperature		102.49±1.17	102.6±1.186
RBS		81.12±13.88	82.32±12.84
Hb		10.40±1.42	9.9±1.60
TLC		13032±4218.97	12304±2665.70
Neutrophil		62.02±13.96	60.96±6.029
Lymphocyte		31.38±12.95	31.1±5.135
Monocyte		5±2.95	5.04±2.58
Eosinophil		1.62±0.89	2.84±1.95
Platelet		2.95±1.07	2.13±0.72
Medical history			
Central nervous system infection		No	No
Metabolic abnormalities		No	Yes

Regarding medical parameters, there was only a slight difference in temperature, with cases having a slightly lower mean temperature (102.49°F) compared to controls (102.6°F). However, when looking at other parameters like random blood sugar (RBS), hemoglobin (Hb), total leukocyte count (TLC), neutrophil count, lymphocyte count, monocyte count, eosinophil count, and platelet count, there was no substantial difference between cases and controls.

Lastly, the findings indicate no cases of CNS infection in either group, but metabolic abnormalities were present in the control group. This suggests that metabolic abnormalities may be a factor worth exploring in this context, given the group differences.

Children with FS (group I) had a significantly higher mean IL-4 level compared to children with fever alone (group II) (Table 2), with a p-value of 0.001. Group II had considerably higher mean IL-6 levels (p<0.001). Patients with FS had their cytokine levels examined in subgroups defined by their family history and the frequency with which they had seizures.

**Table 2:** Mean ± SD level of IL-4 and IL-6 in the two groups pg/mL: picograms per milliliter, IL: interleukin, p-value: probability value <0.05, 0.01, and 0.001 denote statistical significance

Interleukin	Group I (cases)	Group II (controls)	p-value
IL-4 pg/mL	292.85±267.65	81.04±100.26	0.001
IL-6 pg/mL	28.00±57.97	93.09±74.88	0.001

According to Table 3, over 80% of those diagnosed with FS had experienced them in their families. While there was a trend toward higher IL-4 and lower IL-6 in those with a family history of the condition, there was not a distinguishable distinction between the two groups (p=0.876 and p=0.143).

**Table 3:** Distribution of cases on the basis of family history and recurrence of FS N: number, p-value: probability value <0.05, 0.01, and 0.001 denote statistical significance, SD: standard deviation, IL: interleukin, FS: febrile seizures

Variables		N (%)	IL-4 (mean±SD)	IL-6 (mean±SD)
Family history	Present	39 (78%)	299.34±273.67	16.00±16.15
	Absent	11 (22%)	269.81±256.27	70.57±113.68
Total cases		50 (100%)		
p-value			0.876	0.143
FS	First attack	32 (64%)	302.98±277.54	33.17±71.67
	Recurrent attack	18 (36%)	274.83±255.84	18.81±13.41
Total cases		50 (100%)		
p-value			0.725	0.279

Children who had an initial FS exhibited levels of neither IL-4 nor IL-6 that were substantially different from those of children who went on to have more FS (36% of patients). Both groups also underwent hematological analyses. The kids in the group I had low red blood cell counts. Children with FS had a significantly lower mean Hb level (9.44 g/dL) than children with fever alone (11.68 g/dL) in both groups.

## Discussion

Children with simple FS often face several significant issues. The sudden onset of seizures during a fever episode can be a terrifying experience for both the child and their caregivers. This can lead to emotional distress and anxiety, not only during the seizure but also afterward, as there is often uncertainty about whether another seizure will occur. Healthcare providers may struggle with the decision of whether to initiate antiepileptic medication in children with FS. The risk of recurrence is relatively high, but most children eventually outgrow these seizures, making the necessity of long-term medication a subject of debate.

In the present research, a significant incidence of sequelae from prolonged FS in youngsters was observed. This illness results from a combination of factors, one of which is cytokines. Straussberg et al. reported fever in the greatest number of kids between the ages of three and four, whereas the majority of patients with FS were less than two years old. Case children were on average 2.73±1.40 years old, whereas control children were 3.58±1.21 years old [7,20]. There was a statistically insignificant gap between the sexes in both categories, with males making up a somewhat larger percentage of the total. The results of this study indicated that FS affected both sexes equally.

IL-4 is a cytokine that helps reduce inflammation. Average IL-4 concentrations in patients were 292.85 pg/mL, whereas in controls were only 81.04 pg/mL, a statistically significant difference. Individuals having FS had greater IL-4 levels than those with fever alone, which makes sense given the potential involvement of cytokine production in the causes of FS. As a protective measure against seizures, cells may increase the production of anti-inflammatory cytokines during an FS [21]. In contrast, the results of another study conducted by Gupta et al. indicated no rise in the levels of IL-4 in the blood of patients with FS. The type of immunological response is greatly influenced by IL-4 [22]. When operating effectively, it controls the humoral element of the immune response, prevents the synthesis of TH1 cytokines by activating T cells and monocytes, and drives the maturation of naive CD4+ T cells. Microglial polarization is altered by IL-4, and it is thought that this is how it promotes CNS healing. IL-4 promotes CNS healing by downregulating the M1 phenotype and upregulating the M2 phenotype. In their research, Shahrokhi et al. found that mutations in the IL-4 gene were connected to FS. They theorized that a person's susceptibility to FS may be affected by differences in the IL-4 gene, which regulates cytokine production. In addition, IL-4 could control acute microglial activation by dampening cytokine production [23]. Insufficient information exists in India on the function of IL-4 in febrile convulsions.

In the current research, case participants had a significantly lower mean level of IL-6 compared to control participants. Cases had a mean value of 28.00 pg/mL, whereas controls had a value of 93.09 pg/mL. As a key pro-inflammatory cytokine, IL-6 can modulate the immune system. Children who suffered from FS were shown to have low amounts of IL-6 in their blood, which provides more evidence that cytokine intake may have a role in the development of this disorder. These searches are consistent with those of Gupta et al. [22].

Children with FS have been demonstrated to have higher levels of pro-inflammatory cytokine than febrile controls; however, our results contradict these previous studies [24-26]. Virta et al. found that the levels of IL-6 in CSF were considerably more significant in those with FS. Seizures in children with fever are associated with either increased cytokines in the blood or CSF or both [27]. Choi et al. found that individuals with FS also had increased levels of the cytokine IL-6. Patients suffering from febrile convulsions also had significantly higher blood levels of other pro-inflammatory cytokines [28]. Previous research has shown substantial variance in blood IL-6 levels; this may be attributed to factors such as genetics, fever etiology, sample time, and geographic location [23].

IL-6 is a cytokine produced during inflammation by T lymphocytes, macrophages, and epithelial cells. Several studies have shown that IL-6 is a cytokine with several roles in organ development, acute-phase responses, inflammation, and immunological responses, among others [13]. In addition, it is strongly linked to heat illness. Its low plasma half-life of 20-60 minutes is due to expression-level regulation [29]. Acute phase responses, hematopoiesis, and immunological reactions are all aided by IL-6, which is rapidly and transiently generated in response to infections and tissue damage. Dysregulated, ongoing production of IL-6 has a detrimental impact on chronic inflammation and autoimmunity, despite the fact that its expression is tightly regulated by transcriptional and posttranscriptional processes [30].

In the current study, the average Hb of group I was 10.40±1.28 gm/dL, whereas in group II, it was 9.9±1.60 gm/dL. Patients with FS were much more likely to relate to the condition (78%). According to the findings of this particular research, one of the most significant risk factors is having a close family who has had a history of FS. Individuals from families with a history of FS did not exhibit a significantly elevated risk of having elevated IL-4 and IL-6 levels. Compared with individuals who had future attacks, those who experienced their first attack did not have substantially different levels of the cytokines IL-4 than those who had previous attacks. Gupta et al. found similar results, reporting no correlation between blood IL-6 levels and the frequency with which FS returned [22]. Patients with repeated FS had lower IL-4 levels than those suffering their first seizure, according to research by Ha et al. [31]. Possible explanations for this discrepancy include differences in the timing of samples taken after febrile convulsions. Only in individuals who had a prior history of FS that had been identified at the hospital was a seizure recurrence verified.

The current study has several limitations, including a relatively small sample size, potential variability in cytokine concentrations due to the time elapsed between fever onset and serum sample collection, and a focus on investigating only two cytokines. Different cytokine levels can provide light on the association between cytokines and FS. The study collected blood and urine samples within a six-hour window from the onset of seizures or as early as possible. However, the timing of sample collection could impact the results as the study's duration is relatively short. Long-term follow-up data could provide valuable insights into the prognosis and outcomes of children with FS. The study focused on cytokine levels in FS, but it may not explore other potential contributing factors or causes.

## Conclusions

In conclusion, this study aimed to investigate cytokine differences in children with FS compared to those with fever only. Blood and urine samples were collected, and various analyses were performed, including hemogram analysis, glucose level measurement, and IL-4 and IL-6 concentration measurement using ELISA. The study found significant differences in cytokine levels between the two groups, suggesting a potential association between cytokines and FS. Additionally, lower levels of IL-6 in case participants suggest a complex role of cytokines in FS, emphasizing the importance of the cytokine network in this condition. However, there is much more research needed in this field as the current study focused on cytokine levels in FS but may not explore other potential contributing factors or causes.
